# *Ixora parviflora* Protects against UVB-Induced Photoaging by Inhibiting the Expression of MMPs, MAP Kinases, and COX-2 and by Promoting Type I Procollagen Synthesis

**DOI:** 10.1155/2012/417346

**Published:** 2011-12-01

**Authors:** Kuo-Ching Wen, Pei-Ching Fan, Shang-Yuan Tsai, I-Chen Shih, Hsiu-Mei Chiang

**Affiliations:** ^1^Department of Cosmecutics, China Medical University, Taichung 404, Taiwan; ^2^School of Pharmacy, China Medical University, Taichung 404, Taiwan

## Abstract

*Ixora parviflora* with high polyphenol content exhibited antioxidant activity and reducing UVB-induced intracellular reactive oxygen species production. In this study, results of the photoaging screening experiments revealed that IPE at 1000 **μ**g/mL reduced the activity of bacterial collagenase by 92.7 ± 4.2% and reduced the activity of elastase by 32.6 ± 1.4%. Therefore, we investigated the mechanisms by which IPE exerts its anti-photoaging activity. IPE at 1 **μ**g/mL led to an increase in type I procollagen expression and increased total collagen synthesis in fibroblasts at 5 **μ**g/mL. We found that IPE inhibited MMP-1, MMP-3, and MMP-9 expression at doses of 1, 5, and 10 **μ**g/mL, respectively, in fibroblasts exposed to UV irradiation (40 mJ/cm^2^). Gelatin zymography assay showed that IPE at 50 **μ**g/mL inhibited MMP-9 secretion/activity in cultured fibroblasts after UVB exposure. In addition, IPE inhibited the phosphorylation of p38, ERK, and JNK induced by UVB. Furthermore, IPE inhibited the UVB-induced expression of Smad7. In addition, IPE at 1 **μ**g/mL inhibited NO production and COX-2 expression in UV-exposed fibroblasts. These findings show that IPE exhibits anti-inflammatory and anti-photoaging activities, indicating that IPE could be a potential anti-aging agent.

## 1. Introduction

Skin is directly exposed to ultraviolet irradiation, which is a prooxidant agent. Skin aging can be divided into two basic processes, intrinsic and extrinsic aging. UV irradiation is the major cause of extrinsic aging, namely, sun exposure or photoaging, which is characterized by severe wrinkling, sagging, and hyperpigmentation [1]. Disorganization, fragmentation, and dispersion of collagen bundles are prominent features of photodamaged human skin. The role of UVB in skin tumor induction has been accepted for decades. UVB irradiation can cause skin damage by indirectly inducing oxidative stress or directly forming pyrimidine dimers and C-T mutation in DNA, which can lead to photoaging and cancer development [2–4].

The most abundant structural protein in skin connective tissue is type I collagen (90% of extracellular matrix in dermis), which is responsible for conferring strength and resiliency [5, 6]. Type I collagen is synthesized primarily by fibroblasts residing within skin connective tissue (dermis). It is synthesized as a soluble precursor, type I procollagen, which is secreted from fibroblasts and proteolytically processed to form insoluble collagen fibers. Loss of the structural integrity of the collagenous extracellular matrix is believed to be primarily responsible for the wrinkled appearance of photodamaged skin. UV irradiation induces the synthesis of matrix metalloproteinases (MMPs), which degrade collagen in the skin. UVB has been shown to induce the overexpression of MMP-1, -3, and -9 in normal human epidermis *in vivo *[7, 8]. Studies have shown that MMP-mediated collagen destruction accounts, in large part, for the connective tissue damage that occurs in photoaging [9]. Under normal physiological conditions, MMPs are regulated by endogenous inhibitors known as tissue inhibitors of metalloproteinases (TIMP), especially TIMP-1 [10–12]; however, UVB disrupts the balance between MMPs and TIMPs [13]. UV stimulates inhibitory Smad (I Smad; Smad 6 and Smad 7) leading to receptor-activated Smad protein (R Smad; Smad 2 and Smad 3) phosphorylation. It will block R Smad complex translocating into the nucleus [14], thereby reducing expression of type I procollagen and elastin [15].

Besides regulating of MMPs and collagen, excessive UV irradiation can cause acute skin inflammation and lead to the development of skin cancer. Prostaglandins (PGs) and nitric oxide (NO) play important roles in the inflammatory process [16]. Prostaglandins are the products of the cyclooxygenase (COX) pathway of arachidonic acid metabolism. UVB induces cyclooxygenase-2 (COX-2) production, which is the rate-limiting enzyme in PGs generation [17]. UV-induced lipid peroxides are also involved in PGs generation, such as PGE2, an important factor in skin inflammation [18, 19]. UV irradiation also upregulates the expression of inducible nitric oxide synthase (iNOS) to produce NO at sufficiently high levels to react with superoxide, leading to the production of peroxynitrite and other reactive oxygen species (ROS) [20, 21]. ROS drive MAP kinase activation (including ERK, JNK, and p38) which recruits c-Fos and c-Jun to the nucleus and NF-*κ*B activation, and the gene related to proinflammatory would be upregulated subsequently [22].

Polyphenols are abundant in fruits, vegetables, green tea, and wine. Polyphenols such as epigallocatechin-3-gallate (EGCG) found in tea have been shown to have photoprotective properties by hampering collagen destruction and collagenase activation [23, 24]. *Ixora parviflora*, a member of the Rubiaceae family of flowering plants, is rich in polyphenols and used as a folk medicine in India [25–27]. In our previous study, *Ixora parviflora* extract (IPE) exhibited ROS scavenging activity and, therefore, may protect skin from photodamage by diminishing UV-induced ROS production [27]. The aim of this study was to investigate the potential mechanisms by which IPE counteracts UVB-induced overexpression of COX and MMPs, the secretion of MMPs, and the reduction in type I procollagen levels.

## 2. Materials and Methods

### 2.1. Materials

Human foreskin fibroblasts and mouse melanoma cells were obtained from the Bioresource Collection and Research Center (Hsinchu, Taiwan). Gelatin, agarose, methanol, dimethyl sulfoxide (DMSO), doxycycline hyclate (DC), calcium chloride (CaCl_2_), propylene glycol (PG), and DL-dithiothreitol were purchased from Sigma-Aldrich Chemicals (St. Louis, Mo, USA). Fetal bovine serum (FBS), penicillin-streptomycin, trypsin-EDTA, and Dulbecco's modified Eagle's medium (DMEM) were purchased from Gibco, Invitrogen (Carlsbad, Calif, USA). Coomassie blue R-250, dibasic sodium phosphate, lgepal CA-630, tris, sodium dodecyl sulfate (SDS) and, 3-(4,5-dimethylthiazol-2-yl)-2,5-diphenyltetrazolium bromide (MTT) were purchased from USB (Cleveland, Ohi, USA). Collagenase, elastase substrate IV, elastase inhibitor I, and porcine elastase were purchased from Calbiochem, Merck (San Diego, Calif, USA). Fluorogenic peptide substrate I was purchased from R&D Systems (Wiesbaden, Germany). Bradford Reagent was supplied by Bio-Rad Laboratories (Hercules, Calif, USA). Donkey anti-goat IgG-HRP, ERK 1 (C-16), JNK1 (G-13), MMP-1 (L-20), MMP-3 (1B4), MMP-9 (6-6B), type I procollagen (N17, sc-8782), p38 (A-12), p-p38 (Thr 180/Tyr 182)-R, p-JNK (Thr 183/Tyr 185), p-ERK 1/2 (Thr 202/Tyr 204), Smad 3 (38Q), and Smad 7 (H-79) were purchased from Santa Cruz Biotechnology, Inc. (Calif, USA). The Sircol-soluble collagen assay kit was obtained from Biocolor Ltd. (UK). Mouse anti-TIMP-1 carboxy-terminal region monoclonal antibody was purchased from Millpore Corporation (USA).

*Ixora parviflora *extract (IPE) and its hydrolysates were prepared as previously described [27]. The total phenolic content of IPE was 26.2 *μ*g GAE/mg (gallic acid equivalent), and the content of chlorogenic acid was 9.7 ± 1.2 mg/g extract. The abbreviation and hydrolytic conditions of *Ixora parviflora *hydrolysates (IPHs) are as follows: IPH1, 1.2 N HCl at 85°C; IPH2, 2.4 N HCl at 85°C; IPH3, 1.2 N HCl at 100°C; IPH4, 2.4 N HCl at 100°C. The IPE and IPHs were stored at −20°C before use.

### 2.2. Screening of MMP Inhibition

#### 2.2.1. Gelatin Digestion Assay

The assay was carried out as previously described [8]. Briefly, agarose solution was prepared in a collagenase buffer with porcine gelatin. Various concentrations of IPE (125, 250, 500, and 1000 *μ*g/mL; 10 *μ*L) and IPH1–IPH4 (1000 *μ*g/mL; 10 *μ*L) dissolved in 50% propylene glycol were incubated with bacterial collagenase-1 in collagenase buffer for 1 h at room temperature. Doxycycline hyclate (100 *μ*g/mL) was used as the positive control, and 50% propylene glycol served as the negative control. The samples were loaded onto paper disks, placed on gelatin-agarose gel, and then incubated. The degree of gelatin digestion in agarose gel was visualized by the Coomassie Blue staining. Following destaining, the area of the light translucent zone over the blue background was determined densitometrically to estimate gelatinase activity.

The inhibition rate of collagenase was calculated by the following equation:

(1) $$\text{Inhibition}\;\left(\text{\%}\right)=\left(\frac{\left(C-B\right)}{\left(A-B\right)}\right)\times 100,$$

where *A* indicates the absorbance without enzyme and sample, *B* indicates the absorbance with enzyme but without sample, and *C* indicates the absorbance with enzyme and sample.

#### 2.2.2. MMP Activity Assays by Fluorescent Gelatin

The assay was performed as previously described [8]. Enzyme activity assays were performed in 50 mM tris buffer (pH 7.4), 0.15 M NaCl, and 10 mM CaCl_2_. Various concentrations of IPE and IPH1–IPH4 (10, 50, 100, and 500 *μ*g/mL) were tested for their ability to inhibit the activity of MMP. Each concentration of IPE and IPH1–IPH4 was incubated with 1 *μ*M substrate at 37°C for 20 h. Fluorescence intensity was measured at 328 nm (excitation) and 393 nm (emission) with a fluorescence reader (Thermo Electron Corporation, Vantaa, Finland). The rate of collagenase inhibition was calculated by the following equation:

(2) $$\text{Inhibition}\;\left(\text{\%}\right)=\left(\frac{A_{\text{control}}-A_{\text{sample}}}{A_{\text{control}}}\right)\times 100,$$

where *A*_control_ is the absorbance of collagenase and substrate without IPE and *A*_sample_ is that with IPE.

### 2.3. Measurement of Elastase Activity

The ability of IPE to inhibit elastase activity was investigated using elastase from porcine pancreases as previously reported with minor modifications [28]. Elastase inhibitor I (250 *μ*M) was used as the positive control, and 50% propylene glycol served as the negative control. Elastase (500 U) was dissolved in 5 mL of 10 mM tris buffer solution (pH 6.0), and 5 mg of elastase substrate IV was dissolved in 5 mL of 100 mM tris buffer solution (pH 8.0). To measure elastase activity, 100 *μ*L of 100 mM tris buffer solution (pH 8.0), 25 *μ*L of elastase substrate IV solution, 50 *μ*L of sample solution, and 25 *μ*L of elastase solution were dispensed into each well of a 96-well plate and then preincubated for 25 min at room temperature. The elastase activity was quantified by measuring light absorbance at 405 nm by ELISA reader (Tecan, Austria). Each assay was carried out in triplicate.

The inhibition rate of elastase was calculated by the following equation:

(3) $$\text{Inhibition}\;\left(\text{\%}\right)=\left(\frac{A_{\text{control at 405 nm}}-A_{\text{sample at 405 nm}}}{A_{\text{control at 405 nm}}}\right)\times 100.$$

### 2.4. Cell Culture

Human foreskin fibroblasts (Hs68) were maintained in DMEM supplemented with 10% FBS, 100 U/mL penicillin, and 100 U/mL streptomycin at 37°C in 5% CO_2_ humidified air. The cells were subcultured following trypsinization, and cells were used in the 20th to 35th passages.

### 2.5. Cell Viability Test

The fibroblasts were plated at a density of 4 × 10^4^ cells/well, HaCaT cells were plated at a density of 1.5 × 10^4^ cells/well, and B16-F0 cells were plated at a density of 8 × 10^4^ cells/well in 24-well plates per 1 mL medium. The cells were allowed to attach overnight and were treated with 1 mL of various concentrations of IPE dissolved in DMEM and DMSO (<0.1%) for 24 h. The cytotoxicity of IPE was evaluated in cells that had been cultured for 4 h using the MTT assay as previously described [8].

### 2.6. UVB Irradiation

Cells were washed twice with PBS covered with PBS, and then exposed to UV irradiation (302 nm, CL-1000 M, UVP, USA). In our preliminary study, a dose of 40 mJ/cm^2^ UVB irradiation was determined to induce MMP and MAP kinase expression without being cytotoxic (data not shown). After UVB irradiation, PBS was replaced with a serum-free medium with or without sample and then incubated for 24 h for assay of total collagen, MMPs, MAP kinases, and Smad activity.

### 2.7. Western Blot Analysis

Cells were harvested and homogenized with lysis buffer (10 mM Na_3_VO_4_, 10 mg/mL PMSF, 10 mg/mL leupeptin and RIPA buffer, pH 7.4). The lysates were then centrifuged, and protein content was determined using the Bradford method. Cell lysates containing equal amounts of total protein (30 *μ*g) were separated by electrophoresis on SDS-polyacrylamide gel and then transferred to a PVDF membrane (Hybond ECL, Amersham Pharmacia Biotech Inc., Piscataway, NJ, USA). Blots were blocked with 5% (w/v) skim milk in TBS buffer (10 mM Tris-HCl, pH 7.5, 150 mM NaCl) containing 0.05% Tween 20 (TBST). The membrane was incubated overnight at 4°C with goat polyclonal antibodies against MMP-1 (1 : 500) and type I procollagen (1 : 500) and mouse polyclonal antibodies against MMP-3 (1 : 500), MMP-9 (1 : 500), ERK (1 : 500), JNK (1 : 500), p38 (1 : 500), p-ERK (1 : 500), p-JNK (1 : 500), p-p38 (1 : 500), TIMP-1 (1 : 500), Smad 3 (1 : 500), and Smad 7 (1 : 500). The membranes were washed with TBST for 40 min. The blot was then incubated with the corresponding anti-immunoglobulin G-horseradish peroxidase conjugates. Immunoreactive proteins were detected with the ECL western blotting detection system (Fujifilm, LAS-4000). Signal strengths were quantified using a densitometric program (Multi Gauge V2.2).

### 2.8. Gelatin Zymography for MMP-9

Cell-free medium (30 *μ*L) after UVB exposure was mixed with tris-glycine SDS sample buffer without reducing agent and electrophoresed. After electrophoresis, the gels were incubated with washing buffer for 30 min at room temperature. The MMP-9 reaction buffer (1 M Tris-HCl, 1 M CaCl_2_, 1% NaN_3_ and 1% Brij-35, pH 7.4) was then added to the gel and incubated at 37°C for 24 h. The gels were stained with 0.05% Coomassie Blue G-250 until proteinase bands were clearly visible in a blue background. The area of light translucent zones over the blue background was determined by a densitometric program (Multi Gauge V2.2) to estimate gelatinase activity.

### 2.9. Measurement of Total Collagen

Total collagen synthesis in fibroblasts after UVB exposure was measured by the Sircol-soluble collagen assay kit (Biocolor Ltd., UK) according to the manufacturer's protocol. Briefly, cell culture medium was mixed with Sircol dye reagent and incubated at room temperature for 30 min. After centrifugation, ice-cold acid-salt washing reagent was added to the precipitate and then centrifuged. The precipitate was dissolved with the Alka reagent, and the absorption was determined at 555 nm (Tecan, Austria).

### 2.10. Nitric Oxide Scavenging

Hs68 cells were seeded at a density of 3 × 10^5^ cell/well in 6-well plates and incubated at 37°C for 24 h. The medium was replaced with 800 *μ*L of various concentrations of IPE (0.2–100 *μ*M) prepared in PBS and then exposed to 40 mJ/cm^2^ UVB. After exposure, the cells were incubated at 37°C for 24 h. Medium (100 *μ*L) was then sampled and mixed with an equal volume of the Griess reagent. The absorbance of the chromophore formed during the diazotization of nitrite with sulphanilamide and subsequent coupling with naphthylethylenediamine was read at 520 nm and compared to the absorbance of standard solutions of the Griess reagent-treated potassium nitrite.

### 2.11. Statistical Analysis

Differences between groups were analyzed by ANOVA followed by the Scheffe test. A *P* value <0.05 was considered to indicate statistical significance.

## 3. Results

### 3.1. Screening of MMP Inhibition

#### 3.1.1. Inhibitory Effect of IPE and IPH on Bacterial Collagenase-1 Assessed by Gelatin Digestion Assay

**Figure 1:**
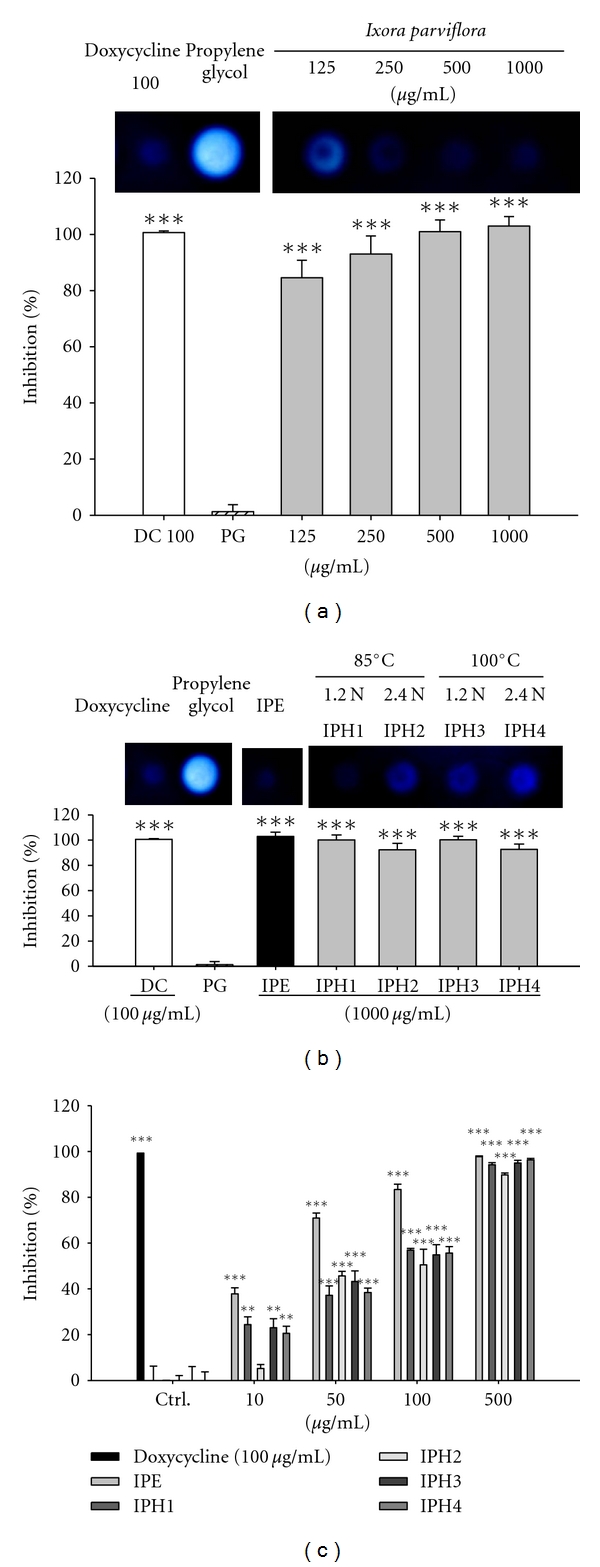
The inhibition of *Ixora parviflora* extract and its hydrolysates on collagenase activity (a, b) and bacterial collagenase activity by fluorometric assay (c). DC: doxycycline; PG: propylene glycol; IPH1: 85°C, 1.2 N HCl; IPH2: 85°C, 2.4 N HCl; IPH3: 100°C, 1.2 N HCl; IPH4: 100°C, 2.4 N HCl. (*n* = 4; **P < 0.01; ***P < 0.001).

For visual investigation and screening of the inhibitory effect of IPE and IPHs on MMP expression, an indirect assay was developed using bacterial collagenase-1 as previously described [8]. As shown in Figure 1(a), the control group treated with reaction products of bacterial collagenase-1 and propylene glycol exhibited the highest gelatinolytic activity in the discrete zone, representing no enzyme inhibition. The inhibition of doxycycline (100 *μ*g/mL, as positive control) was 100.4 ± 0.3%. Following incubation of bacterial collagenase-1 with various concentrations of IPE and IPHs, the inhibition of enzyme activity was compared with enzyme activity in the control group. Gelatinolytic activity decreased in a dose-dependent manner following treatment with IPE. The inhibitory effect of IPE on gelatin digestion by collagenase is shown in Figure 1(b). The rates of collagenase-1 inhibition were 100.2 ± 3.9% for IPH1, 92.4 ± 5.1% for IPH2, 100.3 ± 2.8% for IPH3, and 92.7 ± 4.2% for IPH4. The concentration of IPH in each assay was 1000 *μ*g/mL.

#### 3.1.2. Fluorometric Analysis of the Inhibitory Effect of IPE and IPH on Bacterial Collagenase-1

Fluorescence-conjugated gelatin was used to measure the inhibitory effect of IPE and IPH on bacterial collagenase-1 protein expression. Fluorescence-conjugated substrate was incubated with bacterial collagenase-1 for 20 h in the presence of different concentrations of IPE, IPH, or doxycycline hyclate (positive control) at 37°C. IPE and IPH exhibited significant dose-dependent inhibitory effects on bacterial collagenase-1. As shown in Figure 1(c), treatment with 500 *μ*g/mL of IPE decreased the activity of bacterial collagenase-1 by 97%. The hydrolysates in high concentrations showed similar inhibitory action against collagenase. The effect of IPE and IPH1, 3, and 4 was similar to that of the positive control at 500 *μ*g/mL.

According to the results of gelatin digestion assay and fluorometric analysis on the inhibition of collagenase, the activity of collagenase of IPE was superior to IPHs; therefore, IPE was used in the following study for the effect and mechanism on antiphotoaging.

### 3.2. The Effect of IPE on Elastase Activity

**Figure 2:**
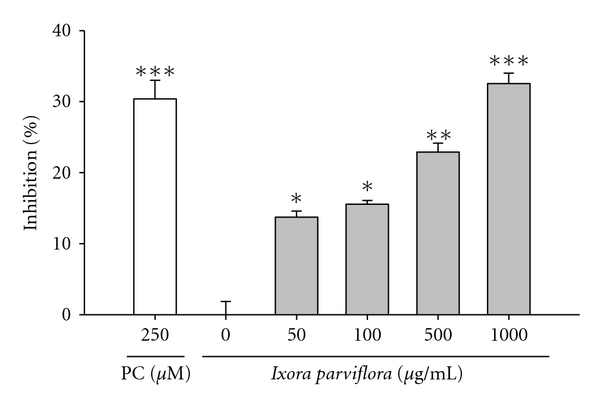
The inhibition of *Ixora parviflora* extract on elastase activity in a dose-dependent manner. (*n* = 4; *P < 0.05; **P < 0.01; ***P < 0.001. PC: positive control, elastase inhibitor I).

This assay measured the synthesis and activity of elastase in cells exposed to IPE. As shown in Figure 2, IPE significantly suppressed elastase activity in a dose-dependent manner. The effect of IPE on reduction of elastase activity was similar to that of the positive control (250 *μ*M elastase inhibitor I) at 500 *μ*g/mL.

### 3.3. Effect of IPE on Cell Viability

**Figure 3:**
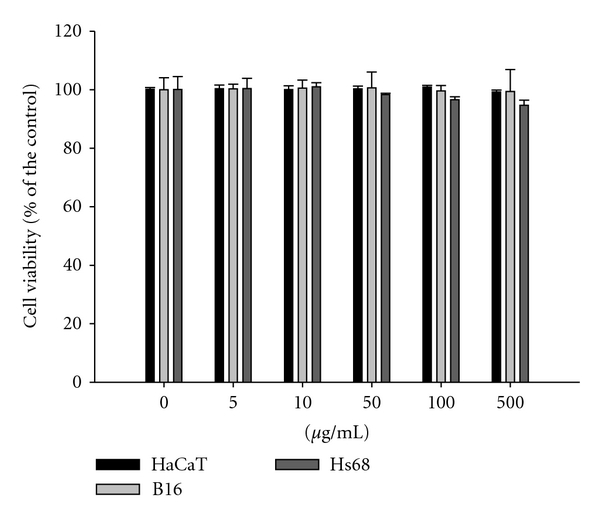
Cell viability (%) of *Ixora parviflora* extract on human keratinocytes (HaCaT), mouse melanoma (B16) and human fibroblasts (Hs68). (*n* = 4).

Hs68, HaCaT, and B16-F0 cells were treated with various concentrations of, IPE and cell viability was measured using the MTT assay. As shown in Figure 3, the survival curve indicates that IPE (5–500 *μ*g/mL) did not exhibit cytotoxic effects on the proliferation of the tested cell lines.

### 3.4. Effects of IPE on UVB-Induced Photoaging

#### 3.4.1. Effect of IPE on Type I Procollagen Expression

**Figure 4:**
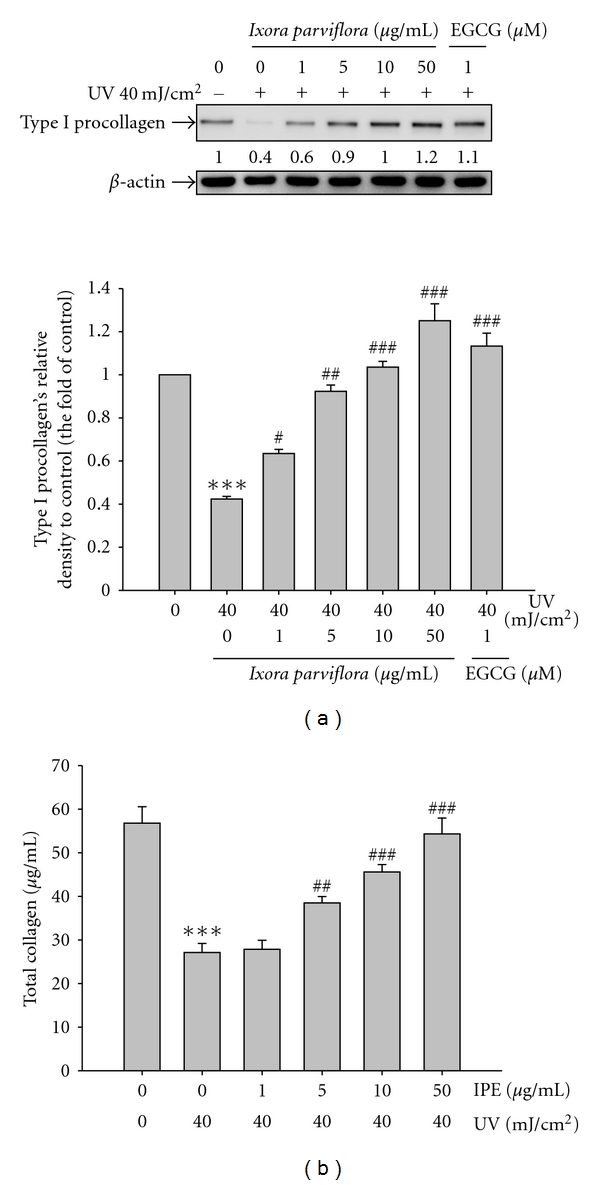
Effect of *Ixora parviflora* extract on the UV-induced type I procollagen expression in human fibroblasts (a) and total collagen synthesis (b). IPE will upregulate type I procollagen expression and synthesis in a dose-dependent manner. (*n* = 4; significant difference versus control (non-UV-exposed): ***P < 0.001. Significant inhibition versus UV-exposed group: ^#^ *P* < 0.05; ^##^ *P* < 0.01; ^###^ *P* < 0.001. EGCG: (−)-epigallocatechin gallate.).

According to our preliminary study, cell viability was greater than 95% relative to that of the control after exposure to 20–80 mJ/cm^2^ UVB irradiation (data not shown). We also found that UV irradiation at a dose of 40 mJ/cm^2^ led to an increase in MMP secretion levels and a decrease in type I procollagen synthesis in UV-exposed fibroblasts (data not shown). This dose is equivalent to about 30 seconds of exposure at noon in the month of July in Central Taiwan as measured by a UV meter (UVP, USA) [28]. Therefore, 40 mJ/cm^2^ was chosen as the exposure dose in the following experiment. Fibroblasts were pretreated with IPE (1–50 *μ*g/mL) for 1 h, exposed to UVB, and then treated with IPE for 48 h. The expression of type I procollagen is shown in Figure 4(a). UVB exposure resulted in 0.4-fold of type I procollagen level relative to the level in control cells; however, after IPE treatment (1–50 *μ*g/mL), type I procollagen levels reached as high as 1.2-fold of those in the control group.

#### 3.4.2. Effect of IPE on Total Collagen Synthesis

Fibroblasts were pretreated with IPE (1–50 *μ*g/mL) for 1 h, exposed to UVB, and then treated with IPE for 24 h. As shown in Figure 4(b), UVB exposure led to a 52.3% reduction in total collagen synthesis. IPE treatment (1, 5, 10, and 50 *μ*g/mL), however, resulted in a dose-dependent restoration of collagen (Figure 4(b)).

#### 3.4.3. Effect of IPE on Smad 3 and 7's Expression

**Figure 5:**
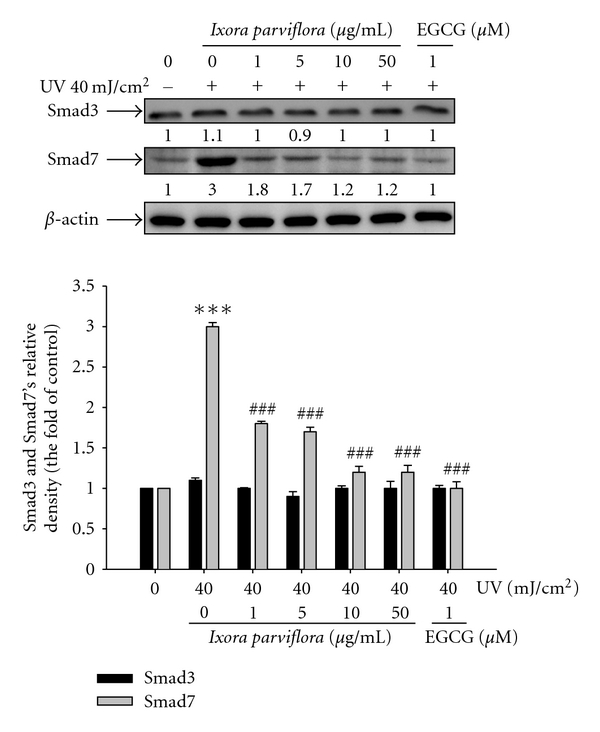
Effect of *Ixora parviflora* extract on the UV-induced Smad 3 and Smad 7 expression in human fibroblasts. (*n* = 4; significant difference versus control (non-UV-exposed): ***P < 0.001. Significant inhibition versus UV-exposed group: ^###^ *P* < 0.001. EGCG: (−)-epigallocatechin gallate.).

As seen in Figure 5, Smad 7 expression was 2-fold higher than that in the control group after UVB irradiation. Smad 7 expression in fibroblasts pretreated with IPE, however, was 1.8-fold higher in cells treated with 1 *μ*g/mL IPE, 1.7-fold higher at a concentration of 5 *μ*g/mL, 1.2-fold higher in cells treated with 10 *μ*g/mL IPE, and 1.2-fold higher in cells exposed to 50 *μ*g/mL IPE relative to the control group.

#### 3.4.4. Effect of IPE on MMPs Expression

**Figure 6:**
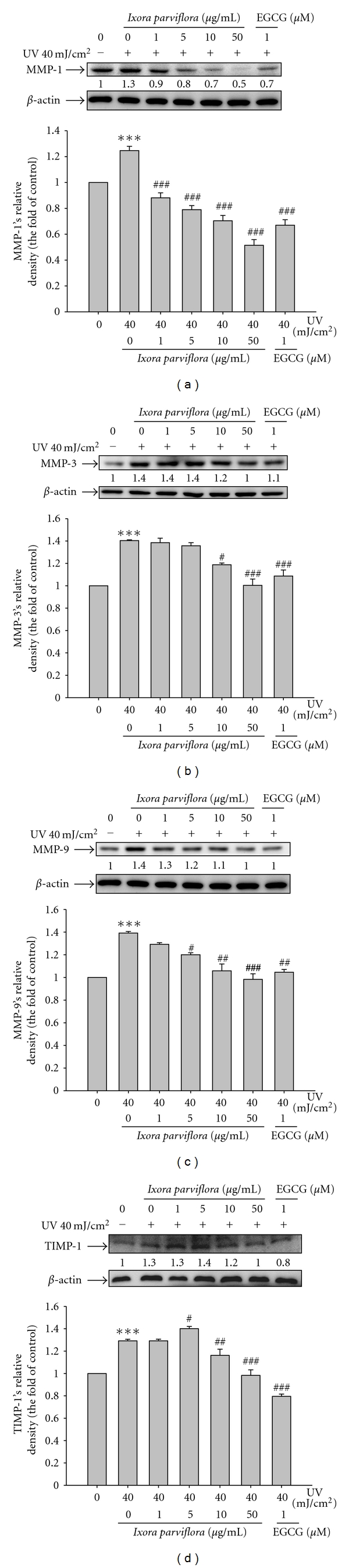
Effect of *Ixora parviflora* extract on the UV-induced MMP-1 (a), MMP-3 (b), MMP-9 (c), and TIMP-1 (d) expression in human fibroblasts. (*n* = 4; significant difference versus control (non-UV-exposed): ***P < 0.001. Significant inhibition versus UV-exposed group: ^#^ *P* < 0.05; ^###^ *P* < 0.001. EGCG: (−)-epigallocatechin gallate.).

Fibroblasts were pretreated with IPE (1–50 *μ*g/mL) for 1 h, exposed to UVB, and then treated with IPE for 48 h. UVB exposure led to a 1.9-fold increase in MMP-1 expression, a 1.4-fold increase in MMP-3 expression, and a 1.4-fold increase in MMP-9 expression (Figures 6(a)–6(c)). IPE treatment, however, had a significant dose-dependent effect on lowering MMP-1 levels. We found that levels of MMP-1 decreased by 29.3% in cells exposed to 1 *μ*g/mL IPE (from 1.3-fold to 0.9-fold of UVB-induced MMP-1 expression), by 36.6% in cells exposed to 5 *μ*g/mL IPE, by 43.5% in cells provided with 10 *μ*g/mL IPE, and by 58.7% in cells exposed to 50 *μ*g/mL IPE treatment (Figure 6(a)). IPE treatment led to similar reductions in MMP-3 and MMP-9 levels (Figures 6(b) and 6(c)).

#### 3.4.5. Effect of IPE on TIMP-1 Expression

TIMP-1 expression was 1.3-fold higher in UVB-irradiated cells than in control cells. Pretreatment of cells with IPE resulted in significant inhibition of TIMP-1 (Figure 6(d)). IPE at concentrations >10 *μ*g/mL significantly suppressed UVB-induced TIMP-1 expression.

### 3.5. Effect of IPE on UVB-Induced MMP-9 Secretion

**Figure 7:**
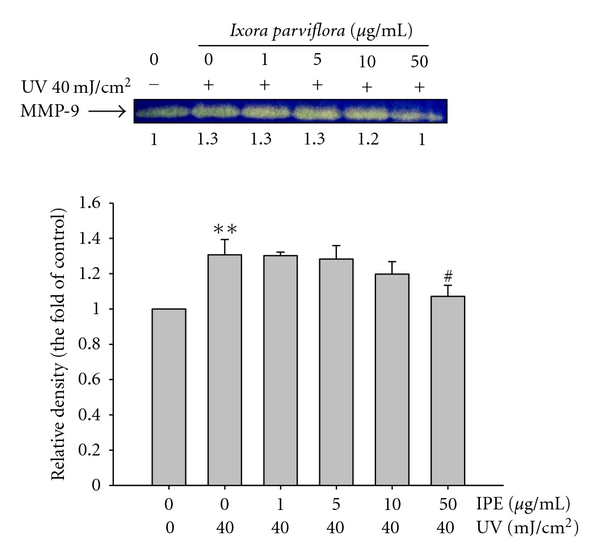
Effect of *Ixora parviflora* extract on MMP-9 by gelatin zymography in the culture medium of human fibroblasts. (*n* = 3; significant difference versus control (non-UV-exposed): **P < 0.01. Significant inhibition versus UV-exposed group: ^#^ *P* < 0.05.).

MMP-9 is a UVB-inducible matrix metalloproteinase that plays an important role in photoaging and skin damage [29–31]. We found that the activity of MMP-9 in fibroblasts exposed to UVB irradiation was 1.3-fold higher than that in control cells; IPE treatment at concentrations of 10 and 50 *μ*g/mL, however, suppressed the activity of MMP-9 in a dose-dependent manner (Figure 7). At concentrations of 50 *μ*g/mL, the level of MMP-9 was lower than that in control cells.

### 3.6. Effect of IPE on MAP Kinase Expression

**Figure 8:**
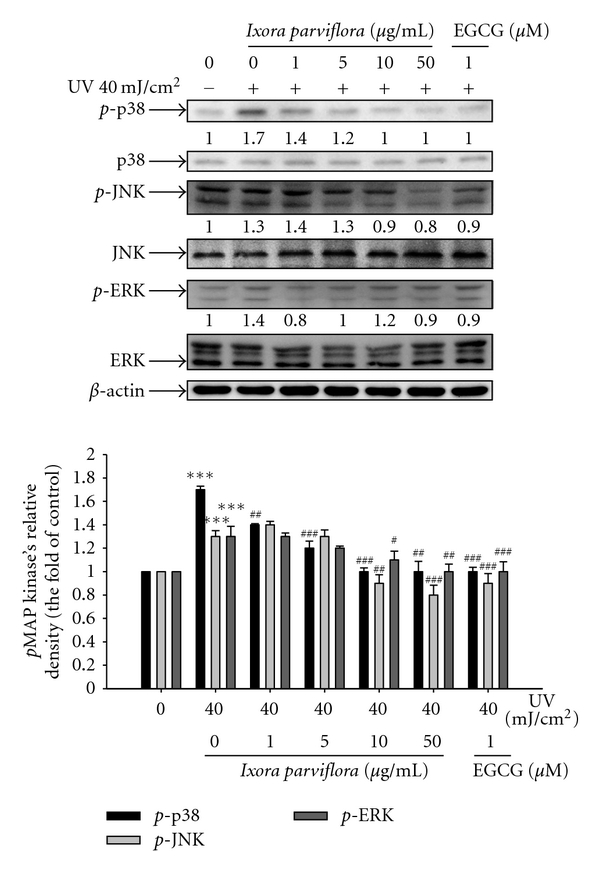
Effect of *Ixora parviflora* extract on the UV-induced phosphorylation of MAP kinase in human fibroblasts. (*n* = 4; Significant difference versus control (non-UV-exposed): ***P < 0.001. significant inhibition versus UV-exposed group: ^#^ *P* < 0.05; ^##^ *P* < 0.01; ^###^ *P* < 0.001. EGCG: (−)-epigallocatechin gallate.).

As shown in Figure 8, UV irradiation at a dose of 40 mJ/cm^2^ resulted in a 1.7-fold increase in levels of phosphorylated p38, a 1.4-fold increase in phosphorylated ERK levels, and a 1.3-fold increase in levels of phosphorylated JNK. IPE treatment (10 *μ*g/mL), however, resulted in a dose-dependent decrease in phosphorylation of the above-mentioned MAP kinases expression.

### 3.7. The Anti-Inflammatory Effect of IPE

#### 3.7.1. Nitric Oxide Scavenging

**Figure 9:**
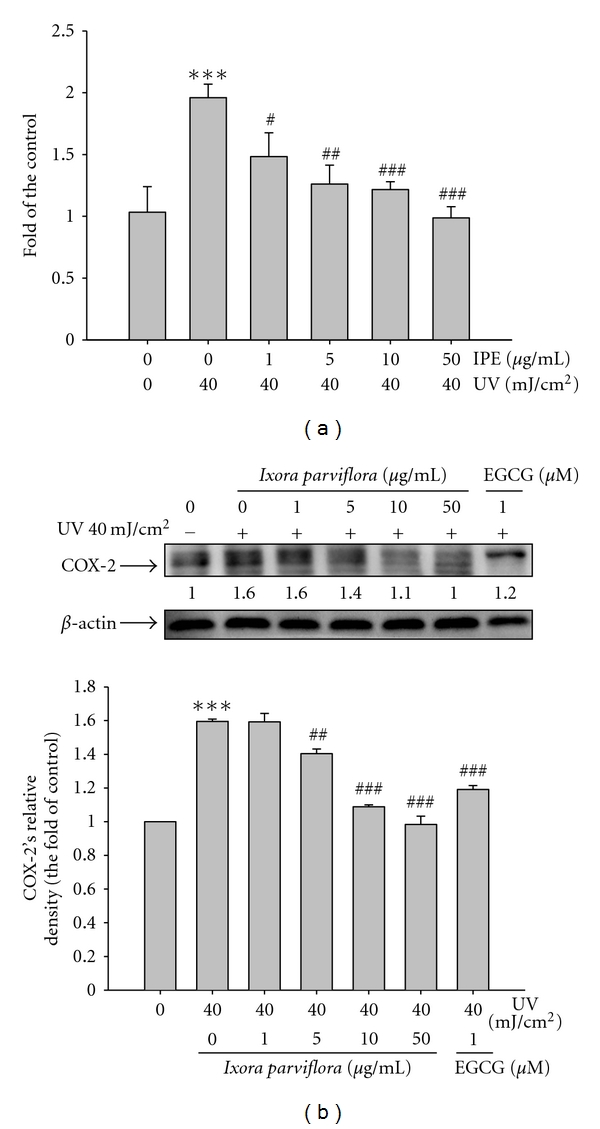
Effect of *Ixora parviflora* extract on NO production in human fibroblasts (a) and the UV-induced expression of COX-2 (b). Human fibroblasts (Hs68) were treated with/without UV 40 mJ/cm^2^ and *Ixora parviflora* extract (IPE) of 1, 5, 10, and 50 *μ*g/mL. (*n* = 3; Significant difference versus control (non-UV-exposed): ***P < 0.001. significant inhibition versus UV-exposed group: ^#^ *P* < 0.05; ^##^ *P* < 0.01; ^###^ *P* < 0.001; EGCG: (−)-epigallocatechin gallate.).

The content of NO in fibroblasts increased to 2-fold after exposure to UVB; however, IPE treatment (1, 5, 10 and 50 *μ*g/mL) significantly reduced the levels of NO (Figure 9(a)).

#### 3.7.2. IPE on COX-2 Expression

The levels of COX-2 were 1.6-fold higher in fibroblasts exposed to UVB (40 mJ/cm^2^) than that in EGCG-exposed control cells (Figure 9(b)). In addition, IPE exhibited a dose-dependent reduction in UVB-induced COX-2 expression. IPE (1, 5, 10, and 50 *μ*g/mL) reduced the UVB-induced expression of COX-2 were 1.6-, 1.4-, 1.1- and 1.0-fold of control, respectively, and the effect was significant at 5 *μ*g/mL. EGCG (1 *μ*M) suppressed UVB-induced COX-2 expression to 1.2-fold of control.

## 4. Discussion

In our previous study, the pH value of IPE in water, methanol, and propylene glycol ranged from 4.9 to 6.7, which is similar to that of skin [27]. Therefore, IPE preparations do not seem to irritate skin.

UV irradiation enhances collagenase activity and contributes to wrinkle formation through degradation of collagen in the dermal extracellular matrix [32, 33]. Collagenase inhibitors have been identified as potential therapeutic agents that can protect against photoaging and wrinkle formation [34]. In the screening experiments, our results indicate that IPE and IPHs inhibit collagenase activity, although the effect of IPE was superior to that of IPHs. The inhibitory activity of IPE at 1000 *μ*g/mL on elastase expression was similar to that of elastase inhibitor I. These findings indicate that IPE may be a potential topical agent to protect against UV-induced skin damage or aging-related disorders. Thus, the IPE was used in following experiments for effects and mechanisms study in Hs68.

Type I collagen is responsible for conferring strength and resiliency and is the most abundant structural protein in skin connective tissue [5]. Upregulation of MMP-1 expression after UV irradiation resulted in the degradation of collagen, the histopathologic hallmark of photoaging [35–37]. It has been shown that quercetin attenuates UV- and H_2_O_2_-induced type I procollagen degradation by reducing JNK/c-Jun activity [38]. Using the Sircol-soluble collagen assay kit, which recognizes the specific secondary structure of collagen ([Gly-X-Y]_n_), we found that IPE treatment (50 *μ*g/mL) restored collagen levels to those of the control. It is well known that UVB irradiation can cause DNA damage and induce MMPs expression such as MMP-1, MMP-3, and MMP-9 resulting in collagen degradation and photoaging [39, 40]. MMP-1 initiates the degradation of types I and III fibrillar collagens [32], MMP-3 activates proMMP-1 to promote type IV collagen [41], and MMP-9 further degrades collagen fragments generated by MMP-1 [42]. MMP-9 is important in the final degradation of collagen. Agents inhibit MMPs expression, and/or activation may prevent photoaging. Natural products exhibited MMPs expression, and/or activity could be used in protection skin from UV damage. Previous studies on pomergranate-derived products inhibited UVB-induced DNA damage, oxidative stress, collagenase (MMP-1), gelatinase (MMP-9), and stromelysin (MMP-3) [39]. *Gynura procumbems* extract inhibited UVB-induced MMP-1 expression and MMP-9 activity [31]. In addition, the high content of polyphenols was shown to be responsible for some of the biological activities observed in those plants. For example, xanthorrhizol in *Curcuma xanthorrhiza *suppressed UVB-induced MMP-1 expression and increased type I procollagen expression [43]. In this study, it was demonstrated that IPE inhibited the UVB-induced overexpression of MMP-1 and MMP-3 as well as the overexpression and secretion/activity of MMP-9 in a dose-dependent manner.

UV irradiation upregulated the phosphorylation of p38, ERK, and JNK, and IPE treatment inhibited phosphorylation of those MAP kinases. *Polypodium leucotomos* extract has been shown to inhibit the activities of MMP-1, -2, -3, and -9 preventing UV damage [44]. In addition, Moon et al. showed that erythrodiol-3-acetate isolated from* Styrax japonica* inhibited UV-induced MMP-1 expression [45]. MAP kinase activation is not only one of the photoaging pathways but is also a factor in MMP production in fibroblasts [22, 46, 47]. In this study, we found that IPE was not only a potent MMP inhibitor but that it also inhibited the MMP-1-initiated degradation of type I collagens. In our previous study, IPE was a potent suppressor of UVB-induced ROS generation [27] and, therefore, might be a promising agent to protect against UVB-induced photodamage. Ho et al. reported that UVB-induced ROS generation promoted downstream signal transduction in human skin fibroblasts [48]. Thus, inhibition of ROS production would help protect skin from photoaging. We speculate that the inhibitory effect of IPE against collagen degradation is most likely due to its antioxidant activity, since the injury to skin caused by UVB may be due to ROS generation.

UV stimulates inhibitory Smad leading to receptor-activated Smad protein phosphorylation causing inhibition of R Smad complex translocating into the nucleus, thereby reducing expression of type I procollagen and elastin [14, 15]. In this study, after UV exposure, IPE treatment resulted in increasing type I procollagen and reducing Smad 7. We expected that the effect on inhibiting of Smad 7 of IPE leading to enhance Smad 2 and Smad 3 phosphorylation may inhibit type I procollagen synthesis. UV irradiation induced the expression of TIMP-1 expression in fibroblasts, and the levels were 1.4-fold higher than control levels in cells exposed to IPE treatment.

UV irradiation leads to direct or indirect DNA damage and the formation of ROS, induces an inflammatory response (induced COX-2 and iNOS), and damages the integrity of the extracellular matrix [49]. Natural products with anti-inflammatory activities can protect DNA from ROS-induced damage. Our studies have shown that IPE possesses antioxidant and anti-inflammatory activities. EGCG has been shown to suppress the UV-induced inflammatory response by inhibiting the activation of NF*κ*B [50]. UV irradiation has been shown to upregulate the expression of inducible nitric oxide synthase (iNOS) to produce NO at sufficiently high levels to react with superoxide, leading to the production of peroxynitrite and other reactive oxygen species. These ROS then induce MAP kinases to produce COX-2 [22]. In this study, we found that the concentration of NO was significantly increased in human fibroblasts that had been exposed to UVB (40 mJ/cm^2^); however, IPE at 1 *μ*g/mL led to a significant reduction in NO, and IPE at 50 *μ*g/mL resulted in NO levels similar to those in control cells. We also found that IPE attenuated the UV-induced overexpression of COX-2. Studies have shown that 1*α*,25-dihydroxyvitamin D3 inhibits UV-induced DNA damage and leads to a reduction in NO levels by inhibiting NOS in keratinocytes [51]. Furthermore, *Porphyra dentate *extract containing catechol, rutin, and hesperidin has been shown to decrease NO production by inhibiting UV-induced NF*κ*B and iNOS expression [52]. In our previous study, IPE exhibited potent ROS scavenging and metal-chelating activities [27]. The scavenging of ROS by IPE may lead to a blockade of the MAP kinase pathway, which would in turn inhibit the activation of NF*κ*B and AP-1 and, hence, inhibit the expression of MMP-1, -3, -9 and COX-2. Inhibition of the MAP kinase pathway, therefore, would prevent the phosphorylation of ERK and the expression of Smad 7, thereby enhancing the expression of type I procollagen.

In summary, our results show that IPE attenuated UVB-induced photodamage and inflammation by modulating the expression of MMPs, MAP kinases, and COX-2. IPE, therefore, appears to be a potent antiphotoaging agent. Further studies on the effect IPE has on other biomarkers in photoaging as well as the identification of the major active components in IPE are warranted.
